# Single-center experience using anakinra for steroid-refractory immune effector cell-associated neurotoxicity syndrome (ICANS)

**DOI:** 10.1136/jitc-2021-003847

**Published:** 2022-01-07

**Authors:** Marc Wehrli, Kathleen Gallagher, Yi-Bin Chen, Mark B Leick, Steven L McAfee, Areej R El-Jawahri, Zachariah DeFilipp, Nora Horick, Paul O'Donnell, Thomas Spitzer, Bimal Dey, Daniella Cook, Michael Trailor, Kevin Lindell, Marcela V Maus, Matthew J Frigault

**Affiliations:** 1 Cellular Immunotherapy Program, Massachusetts General Hospital Cancer Center and Harvard Medical School, Boston, Massachusetts, USA; 2 Harvard Medical School, Boston, MA, USA; 3 Hematopoietic Cell Transplant & Cell Therapy Program, Massachusetts General Hospital Cancer Center and Harvard Medical School, Boston, MA, USA

**Keywords:** chimeric antigen receptors, inflammation, cytokines, drug therapy, combination

## Abstract

In addition to remarkable antitumor activity, chimeric antigen receptor (CAR) T-cell therapy is associated with acute toxicities such as cytokine release syndrome (CRS) and immune effector cell-associated neurotoxicity syndrome (ICANS). Current treatment guidelines for CRS and ICANS include use of tocilizumab, a monoclonal antibody that blocks the interleukin (IL)-6 receptor, and corticosteroids. In patients with refractory CRS, use of several other agents as third-line therapy (including siltuximab, ruxolitinib, anakinra, dasatinib, and cyclophosphamide) has been reported on an anecdotal basis. At our institution, anakinra has become the standard treatment for the management of steroid-refractory ICANS with or without CRS, based on recent animal data demonstrating the role of IL-1 in the pathogenesis of ICANS/CRS. Here, we retrospectively analyzed clinical and laboratory parameters, including serum cytokines, in 14 patients at our center treated with anakinra for steroid-refractory ICANS with or without CRS after standard treatment with tisagenlecleucel (Kymriah) or axicabtagene ciloleucel (Yescarta) CD19-targeting CAR T. We observed statistically significant and rapid reductions in fever, inflammatory cytokines, and biomarkers associated with ICANS/CRS after anakinra treatment. With three daily subcutaneous doses, anakinra did not have a clear, clinically dramatic effect on neurotoxicity, and its use did not result in rapid tapering of corticosteroids; although neutropenia and thrombocytopenia were common at the time of anakinra dosing, there were no clear delays in hematopoietic recovery or infections that were directly attributable to anakinra. Anakinra may be useful adjunct to steroids and tocilizumab in the management of CRS and/or steroid-refractory ICANs resulting from CAR T-cell therapies, but prospective studies are needed to determine its efficacy in these settings.

## Background

Chimeric antigen receptor (CAR) T cells have revolutionized the landscape of treatment options for certain hematological malignancies. Currently, there are four autologous CAR T cell products targeting CD19 that are approved by the US Food and Drug Administration (FDA). Tisagenlecleucel (Kymriah), axicabtagene ciloleucel (Yescarta), brexucabtagene autoleucel (Tecartus), and lisocabtagene maraleucel (Breyanzi) have demonstrated durable remissions in patients with relapsed and refractory B-cell malignancies.^1–4^ With the growing number of cellular therapy studies targeting novel, non-CD19 antigens, we have observed that cytokine release syndrome (CRS) and immune effector cell-associated neurotoxicity syndrome (ICANS) are likely class effects of immune effector cell therapies and, as such, will likely necessitate additional management strategies for expansion to broader patient populations.

Despite the success of CD19-targeting CAR T-cell therapy, ICANS and CRS continue to present barriers to wider patient access. Traditional management of CRS involves the use of tocilizumab, an interleukin (IL)-6R antagonist and corticosteroids. Based on the pivotal, phase II studies that led to the approval of all four commercially available products, grade 3 or higher severe CRS/ICANS was observed in upwards of 30% of patients despite aggressive management with tocilizumab and steroids.^1–3^ Additionally, recently presented data using early administration of prophylactic steroids for CRS/ICANS prevention still demonstrated a 13% severe ICANS rate.^5^ We have previously demonstrated that refractory CRS/ICANS has been associated with increased healthcare resource use, as well as increased treatment-related mortality, suggesting that improved management strategies will likely increase patient access as well as improve long-term outcomes.^6 7^ Recent animal data have suggested a critical role for IL-1 signaling in the pathogenesis of ICANS and CRS. Anakinra, an IL-1R antagonist, is FDA approved as a subcutaneous injection for rheumatoid arthritis and cryopyrin-associated periodic syndromes, and has been considered for use in refractory CRS/ICANS.^8^ As such, recent consensus guidelines from the Society for Immunotherapy of Cancer have suggested anakinra as a third-line agent for refractory CAR T-associated toxicities.^9^ More importantly, although tocilizumab has shown benefit in the management of CRS, it does not improve ICANS due to poor penetration of the blood–brain barrier^10 11^; furthermore, because circulating IL-6 is cleared via internalization through its receptor, blockade of the IL-6R may paradoxically expose the brain to higher levels of IL-6. Based on this rationale, and in the absence of prospective or retrospective data describing the use of anakinra in the setting of CAR T cell-related CRS or ICANS, our institution has been empirically using anakinra as a ‘third-line’ agent in cases of tocilizumab-refractory CRS or steroid-refractory ICANS. We retrospectively analyzed our patient population that was treated empirically with anakinra as a rescue agent for refractory CRS or ICANS after receiving tisagenlecleucel or axicabtagene ciloleucel at our institution. We report on the clinical profiles of these patients, in which we found that we were primarily using anakinra as a second-line agent for refractory ICANS. We also evaluated clinical markers of CRS (temperature, CRP, and ferritin), cytopenias, and performed testing for a panel of cytokines from banked serum samples that were collected in the course of their care.

## Materials and methods

### Patients

Patients were treated between 2017 and 2020 at the Massachusetts General Hospital (MGH) Cancer Center. All patients included were diagnosed with CD19-positive hematological malignancies. The use of anakinra (daily 100–200 mg subcutaneously for up to 3 days) was based on the physician’s discretion for the management of corticosteroid-refractory ICANS with or without CRS being refractory to tocilizumab and/or corticosteroids. Corticosteroid-refractory ICANS was defined as any patient who did not show clinical signs of improvement after at least one dose of corticosteroids within at least 1 day (median 24 hours) for axi-cel, or within the same day for tisa-cel. ICANS grading is based on notes from our patient information system and was not part of a clinical trial. Therefore, formal ICE scores were not always available. The time point of data cut-off was October 1, 2020.

### Cytokine analysis using Luminex multiplex system

Out of the 14 patients in this study, 13 patient’s frozen serum samples were stored at the Immune Monitoring Laboratory (IML) of the MGH Cancer Center. Patients without all time points preserved (4 of 13) were excluded from the study. Baseline samples were collected on the day of CAR T infusion (before infusion of CAR T cells). Serum samples from nine patients treated with anakinra were analyzed by the custom 27-plex Luminex assay panel (R and D Systems) to detect soluble proteins in patient serum. Samples were analyzed on the FLEXMAP 3D instrument. This analysis included CCL2/MCP-1, CCL3/MIP-1 alpha, CCL4/MIP-1 beta, CD25/IL-2R alpha, CXCL10/IP-10/CRG-2, GM-CSF, IL-1 alpha/IL-1F1, IL-1 beta/IL-1F2, IL-1ra/IL-1F3, IL-2, IL-6, IL-7, IL-15, IL-18/IL-1F4, IL-6R alpha, tumor necrosis factor-alpha and IL-10. The Luminex quantifies multiple analytes in one well. Briefly, analyte-specific antibodies have been precoated onto magnetic beads embedded with fluorophores at set ratios for each unique bead region. Beads, standards, and samples were pipetted into wells, and the immobilized antibodies bound the analyte of interest. Patient samples were performed in duplicate and diluted in a 1:2 ratio. Extrapolated maximal values were adapted to the highest amount of the corresponding standard of the assay. Cytokine fold change over baseline was calculated as follows: cytokine value (pg/mL) before or after anakinra/baseline (cytokine value (pg/mL).

### Statistical analysis

Statistical analysis was performed as indicated in the figure legends using GraphPad Prism V.9 (GraphPad Software, San Diego, California, USA). Percentage change was calculated as follows: percentage change (%)=((value after anakinra−value before anakinra)/value before anakinra))×100. P values (two-tailed) of less than 0.05 were considered as statistically significant.

## Results

### Patient characteristics and ICANS/CRS management

**Figure 1:**
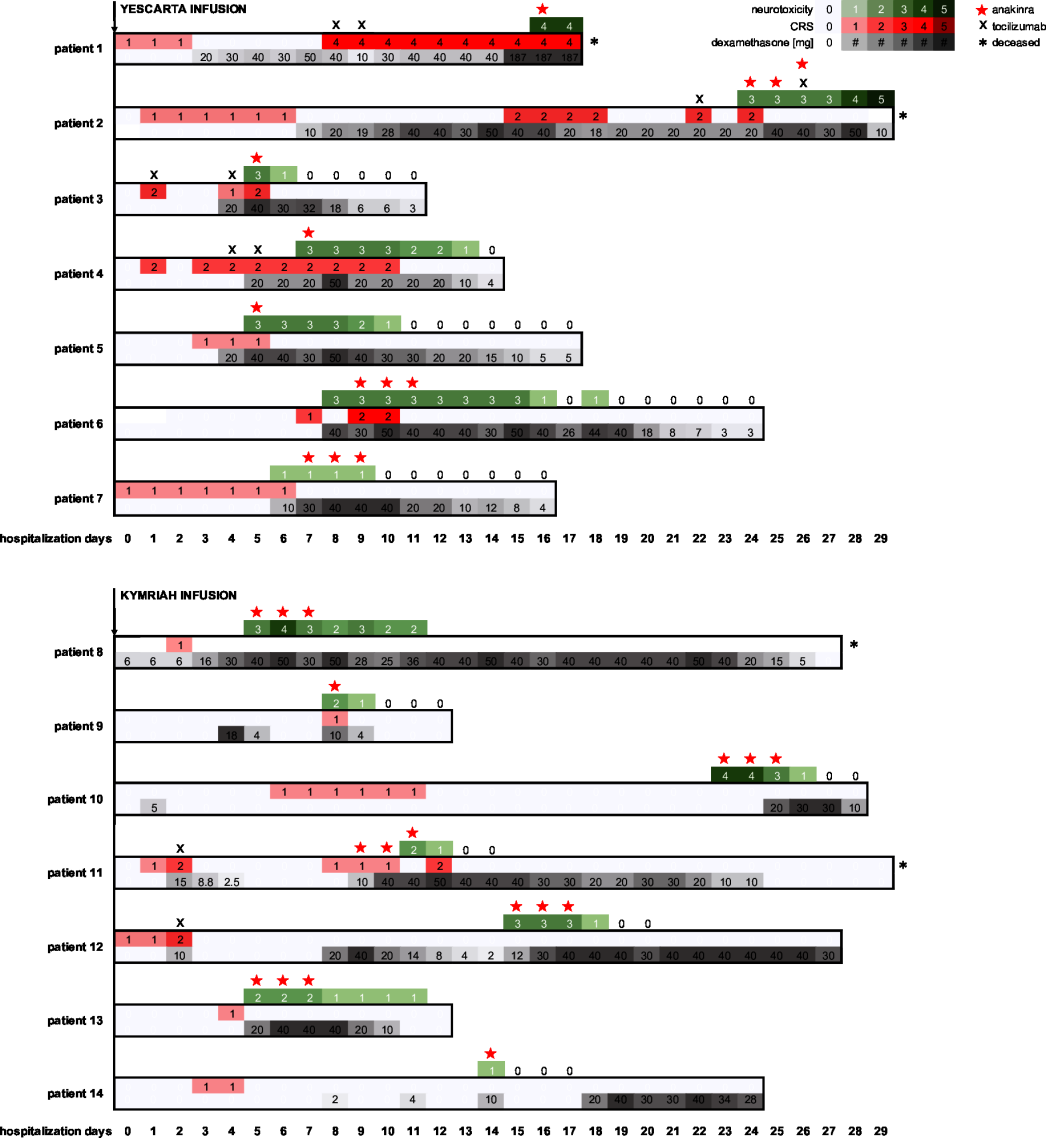
Swimmer’s plot summarizing clinical course with CRS, peak ICANS, and corresponding treatment for each patient. CRS was graded according to autologous stem cell transplantation consensus grading 2020. ICANS was graded retrospectively based on clinical notes from the patient information system. Corticosteroid application is indicated in milligram in gray graduated boxes. Time period represents hospitalization days starting at CART infusion. The graph is subdivided into axicabtagene ciloleucel (Yescarta) and tisagenlecleucel (Kymriah). * indicates deceased; X indicates application of tocilizumab; star indicates application of anakinra. CRS, cytokine release syndrome; ICANS, immune effector cell-associated neurotoxicity syndrome.

In our retrospective analysis, 14 patients with CD19-positive hematological malignancies treated with CD19-directed CAR T-cell therapy developed CRS or ICANS that was refractory to tocilizumab and/or corticosteroids (table 1 and figure 1). The median age of patients at the time of CAR T therapy was 58 with a range of 26–75 years. Of the 14 patients, 6 were female. The median Eastern Cooperative Oncology Group performance status prior to starting lymphodepleting chemotherapy was 1 (range 0–2). Of the 14 patients, 6 were diagnosed with diffuse large B-cell lymphoma (DLBCL) and 5 had central nervous system (CNS) involvement. Besides DLBCL, three had transformed follicular lymphoma (tFL), two had mantle cell lymphoma, one had lymphoblastic lymphoma, onehad Burkitt’s lymphoma and one had primary mediastinal B-cell lymphoma. The median peak ICANS and peak CRS were three and two, respectively. The median peak LDH value was 524 U/L with a range from 163 to 17 624 U/L. The median number of previous lines of treatment was 4 (range of 2–8 treatment lines), with 2 patients having been treated with prior autologous stem cell transplant. Out of 14 patients, 7 were treated with axicabtagene ciloleucel and 7 were treated with tisagenlecleucel. Systemic corticosteroids were given starting a median of 4.5 days (range 1–25 days) after CAR T infusion and for a median of 12.5 cumulative days (range 3–26 days) and a median cumulative dose of 297 mg dexamethasone (range 26–1228 mg). Anakinra was initiated at a median of 8.5 days after CAR T infusion following failure of corticosteroids with a median of three doses given (range 1–3 times) at a dose range of 100–200 mg/day subcutaneously. In the patient population of corticosteroid-refractory ICANS with CRS of grade 2 or higher, the median number of tocilizumab doses given was 2 (range 1–3 doses) starting at a median of 4 days (range 1–12 days) after CAR T-cell infusion (table 1). All of the patients received anakinra to treat steroid-refractory ICANS with or without CRS, rather than simply tocilizumab-refractory CRS without ICANS (figure 1). In 9 out of 14 patients, the reduction of peak ICANS or refractory CRS or could be reached within 1 day after the last anakinra dose. One patient (patient 2) died because of refractory neurotoxicity on day 29 after CAR T cell infusion, despite the use of tocilizumab, corticosteroids, and anakinra. Three patients died of multiple infections including coinfections with bacteria and fungi. Routine prophylaxis for patients receiving CAR T cells consisted of an antiviral (such as famciclovir) and atovaquone, and fluconazole was added for patients receiving more than a single day of steroids. Antibiotics were based on standard protocols for febrile neutropenia and on culture data. There was one case where anakinra was initiated before ICANS as the initial management in effort to spare a prolonged course of corticosteroids. However, corticosteroids were added due to insufficient improvement 48 hours later. There were two patients who had grade 1 ICANS when anakinra and corticosteroids were started. This procedure was initiated due to the prolonged CRS or neurotoxicity in these subjects. Six patients died due to progression of disease.

**Table 1:** Patient characteristics with disease type, laboratory parameters, CART cell product including previous therapy, and toxicity treatment

	Count	Median	Range
Age (years) at the time of CART infusion		58	26–75 years
Sex (F:M)	6:8		
ECOG performance status		1	0–2
Ann Arbor stage		IV	I–IV
Disease type			
DLBCL	6 patients		
DLBCL with CNS involvement	3 of 6 patients		
tFL	3 patients		
MCL with CNS involvement	2 patients		
Lymphoblastic lymphoma	1 patient		
Burkitt lymphoma	1 patient		
PMBCL	1 patient		
Peak ICANS		3	1–5
Peak CRS		2	1–4
Peak LDH		524 U/L	163–17624 U/L (norm 110–220 U/L)
CART cell product			
Axicabtagene ciloleucel—Yescarta	7 patients		
Tisagenlecleucel—Kymriah	7 patients		
Previous treatment			
Previous treatment lines		4	2–8
Previous ASCT	2 patients	0	
Hospitalization days		20.5 days	11–29 days
Toxicity treatment			
Corticosteroid start (after CART infusion)	14 of 14	4.5 days	1–25 days
Cumulative dexamethasone dose	14 of 14	297 mg	26–1228 mg
Cumulative dexamethasone days	14 of 14	12.5 days	3–26 days
Anakinra start (after CART infusion)	14 of 14	8.5 days	5–23 days
Anakinra dose (100–200 mg subcutaneous)	14 of 14	3 times	1–3 times
Number of patients with CRS grade ≥2	7 of 14		
Number of patients treated with tocilizumab:	7 of 14		
Tocilizumab start (after CART infusion)	7 of 7	4 days	1–12 days
Tocilizumab dose (times 8 mg/kg)	7 of 7	2 times	1–3 times
Survival			
Patients alive (%)	29		
Patients alive (n)	4		
Patients deceased (%)	71		
Day of death		29th day	18–198 days
Cause of death	6 of 10 PDs		
	3 of 10 Infections*		
	1 of 10 ICANS 5		
Infections*			
Fungal infection			
1 patient: mucormycosis			
Coinfection of fungi and bacteria:			
1 patient: *Candida albicans* and *Pseudomonas aeruginosa*			
1 patient: *Enterococcus faecalis* and fungal infection based on 1–3 beta glucan test			

Hospitalization days from CART infusion (day 0) to discharge are indicated.

ASCT, autologous stem cell transplantation; CNS, central nervous system; CRS, cytokine release syndrome; DLBCL, diffuse large B-cell lymphoma; ECOG, Eastern Cooperative Oncology Group; ICANS, immune effector cell-associated neurotoxicity syndrome; LDH, lactate dehydrogenase; MCL, mantle cell lymphoma; PD, progressive disease; PMBCL, primary mediastinal B-cell lymphoma; tFL, transformed follicular lymphoma.

### Temperature and laboratory parameters of toxicity in anakinra-treated patients

**Figure 2:**
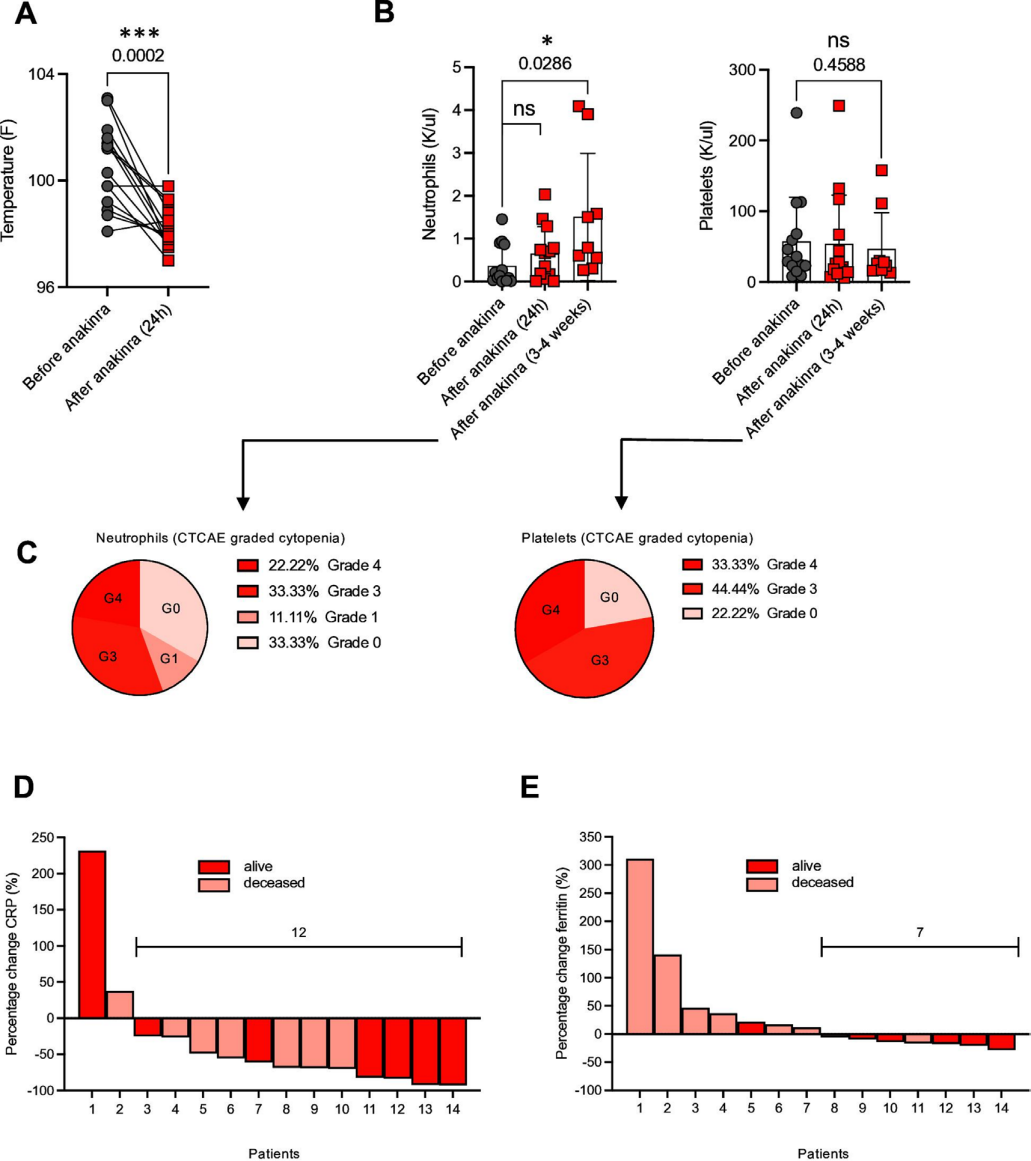
Temperature and laboratory variables as parameters of toxicity in anakinra-treated patients. (A) Temperature: highest value 24 hours before/after the first/last anakinra dose. (B) Neutrophil and platelet count before and after anakinra. Each dot and square connected by a line indicate one patient, n=14. Temperature: paired t test. Neutrophil count: paired t test. Platelet count: paired t test, p value=0.4588 (ns). Pie charts of neutropenia and thrombopenia 3–4 weeks after anakinra application. Grading (G 0–4) in accordance with the Common Terminology Criteria for Adverse Events (CTCAE) V.5 (C). Waterfall plots of percentage change of CRP (D) and ferritin (E). Each column represents a patient. Inflammatory markers were obtained within the 48 hours before the first dose and after the last anakinra dose. Dark red indicates patients alive at the time of data cut. Light red indicates patients deceased. Statistical analysis was performed using GraphPad Prism V.9.0 (GraphPad software, LLC). *P=0.0286, ***P=0.0002. CRP, C reactive protein; ns, not significant.

The median temperature at the time of first anakinra dose was 100.8°F. Maximum temperature was significantly reduced 24 hours after the last dose of anakinra (figure 2A). Whereas the effect of anakinra on temperature was faster and more quantitative, the effect of anakinra on neurotoxicity was not as immediate or dramatic (figure 1). A significant increase of neutrophil counts was observed between 22 and 32 days after treatment with anakinra (figure 2B, left). Although there is no formal comparator cohort in our study, recovery of neutropenia within 3–4 weeks after CAR T-cell infusion is not atypical for this patient population. Still 55.55% of patients had grade 3 or 4 neutropenia 3–4 weeks after the treatment with anakinra (figure 2C, left). Continued thrombocytopenia, with a median platelet count of 26 K/uL (range from 13 to 158 K/uL) was observed (figure 2B, right). We observed that 77.77% of patients had grade 3 or 4 thrombocytopenia 3–4 weeks after anakinra (figure 2C, right). Anakinra did reduce the levels of clinical inflammatory markers such as C reactive protein (CRP), which was decreased in 12 out of 14 patients, and ferritin, which was reduced in 7 out of 14 patients (figure 2D, E).

### Serum biomarkers of toxicity in patients treated with third-line anakinra

**Figure 3:**
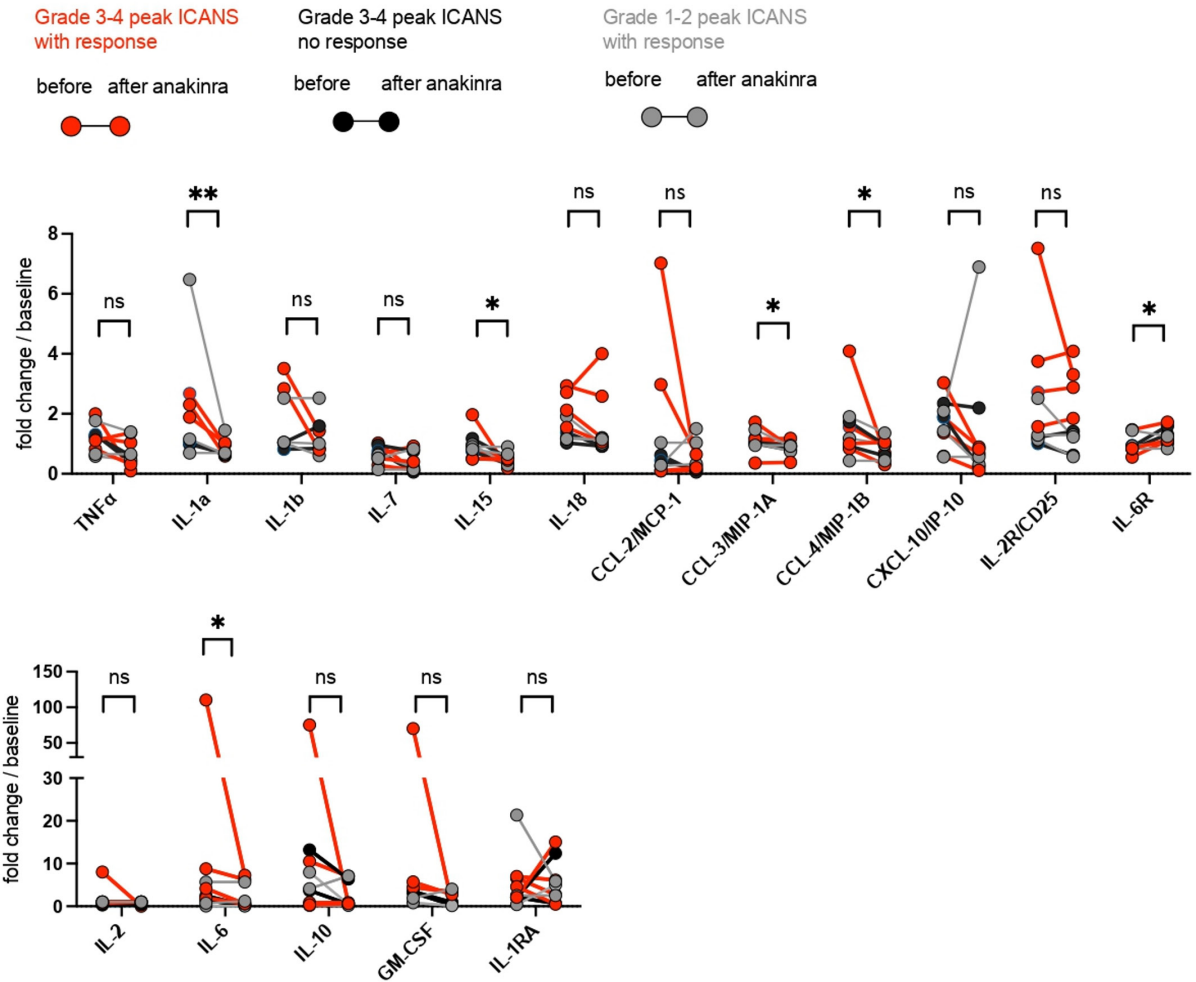
Analysis of inflammatory cytokines before and after anakinra application. Analysis of cytokines in patient’s serum before and after anakinra application. Samples were collected within 1–2 days (median 1 day) before and 2–8 days (median 4 days) after the last anakinra dose (corresponding to days 3, 7, 14, 21, and 28 after CART infusion). (A) The custom 27-plex Luminex assay panel was used. Each dot connected by a line indicates one patient. Peak ICANS grades 3–4 with subsequent neurotoxicity reduction (response) are indicated by red circles. Peak ICANS grades 3–4 without subsequent neurotoxicity reduction (no response) are indicated by black circles. Grade 1–2 ICANS are indicated by gray circles, n=9. Ratio paired t-test. Statistical analysis was performed using GraphPad Prism V.9.0 (GraphPad Software, LLC.). *P<0.05, **P<0.01. ICANS, immune effector cell-associated neurotoxicity syndrome; ns, not significant.

Cytokines were analyzed from frozen serum samples using a 27-plex Luminex assay panel. Due to the retrospective nature of the study, samples were collected within 2–8 days (median 4 days) after the last anakinra dose (corresponding to day 3, 7, 14, 21, and 28 after CART infusion) and have been compared with samples prior to initiation of anakinra. A significant reduction of the inflammatory cytokines IL-1a, IL-15, CCL-3, CCL-4, and IL-6 was observed after the administration of anakinra in this patient population (figure 3) as well as a significant increase of IL-6R in this patient cohort. Patients with peak ICANS grade 3 and 4 were separated into two groups: one with neurotoxicity reduction after anakinra (figure 3; red circles, anakinra response) and another group without neurotoxicity reduction after anakinra (figure 3; black circles, no anakinra response). The remaining patients consisted of peak grade 1–2 ICANS with neurotoxicity reduction after anakinra (figure 3; gray circles, anakinra response). A trend toward a more pronounced reduction of IL-1a, IL-1b, CCL-4, CXCL-10, and IL-6 could be observed in patients with peak ICANS 3–4 and a clinical response after treatment with anakinra. Such an effect could not be detected in the patients who had grades 3–4 ICANS but did not have improvements in neurotoxicity after anakinra.

### Clinical outcomes of patients with refractory CRS/ICANS treated with anakinra

With a median follow-up of 23.7 months, the best overall response rate was 55% (95% CI 23% to 83%). Although the median overall survival (OS) of these 14 patients was 2.8 months, the OS of 6 and 12 months was 36% and 29%, respectively, with 50% of responders maintaining their response at time of data cut.

## Discussion

Tocilizumab and steroid-refractory ICANS and CRS remain key barriers to the optimal management of patients with CAR T. Additionally, although steroids have shown efficacy in the management of ICANS, some reports suggest that tocilizumab may precipitate ICANS in high-risk patients due to its inability to penetrate the blood–brain barrier and transient increase in systemic and CNS IL-6 levels following tocilizumab administration.^2 4 10–12^ Therefore, corticosteroids remain a key element for the management of ICANS. Although some evidence suggests that prolonged steroid use is associated with inferior clinical outcomes, it is still controversial as to whether steroid use is causative, or rather, a surrogate of poor outcomes in a high-risk patient population.^6 9 13^

As an alternative to corticosteroids, anakinra is one of several steroid-sparing agents which has been suggested for the management of CRS and ICANS. Anakinra is a recombinant humanized IL-1 receptor antagonist, with a relatively short half-life of 4–6 hours, that has demonstrated efficacy in managing CRS and ICANS in humanized mouse models.^14 15^ IL-1 blocking therapies are already approved for the treatment of rare autoinflammatory diseases associated with periodic fevers and are used as off-label treatment for secondary hemophagocytic lymphohistiocytosis/macrophage activation syndrome, a syndrome hypothesized to share a common pathophysiology with CRS via excess T-cell and macrophage activation.^13 16 17^ Moreover, anakinra has been studied extensively in the setting of sepsis and stroke at doses significantly higher than those used here without notable toxicities.^16 18^

Here we present a single-center, retrospective study of 14 patients who received axicabtagene ciloleucel or tisagenlecleucel and developed corticosteroid-refractory ICANS with or without CRS that refractory to tocilizumab and corticosteroids. In this patient population, we observed rapid improvement of CRS, including fever and clinical correlates of toxicity such as CRP and ferritin following the use of anakinra. Reductions in CRP and ferritin following anakinra treatment have also been observed in patients with COVID-19-associated acute respiratory distress syndrome, a potentially similar cytokine-mediated hyperinflammatory syndrome.^19^ Interestingly, Strati and colleagues recently reported similar findings by demonstrating a reduction of the CRP area under the curve in patients with CAR T-cell therapy-associated toxicity who responded to anakinra.^13^

Neutropenia and thrombocytopenia are frequently observed after CAR T-cell treatment, particularly in patients with severe CRS, and one potential concern with the use of anakinra is potential for further delay in hematopoietic recovery. We observed significantly improved neutrophil counts compared with platelet counts 3–4 weeks after anakinra treatment. Unfortunately, we do not have a comparator arm here, but we note that this timeline is generally similar to other published studies with CAR T cells.^20 21^ In general, neutropenia has been described in <2% in a placebo-controlled trial with patients treated with anakinra.^22^ Although there is no formal comparator cohort in our study, it appears that anakinra does not have a significant impact on hematopoietic recovery when used to manage refractory CRS or neurotoxicity. In addition to clinical markers of inflammation, we also found statistically significant declines in cytokines correlated with severity of CRS and ICANS including IL-1a, IL-15, CCL-3, CCL-4, and IL-6.^11 23 24^ Even though statistically not significant, a trend toward a more pronounced reduction of inflammatory cytokines could be observed in patients with peak ICANS 3–4 and a clinical response to anakinra, in comparison to those patients with ICANS 3–4 without a clinical response to anakinra. Due to interpatient differences of absolute cytokine values, we decided to graph fold increase instead of absolute values in order to better visualize the data. As many of the patients had previously received prior treatment with tocilizumab and/or steroids, and continued on such therapy, it remains to be determined whether the combination of tocilizumab, steroids, and/or anakinra led to the reduction of the aforementioned inflammatory cytokines. Moreover, the sample size and the concomitant medication make it difficult to determine conclusively whether anakinra increased infection risk. Unfortunately, the use of anakinra did not immediately enable tapering or withdrawal of corticosteroids in most cases.

It is more difficult to attribute a reduction in neurotoxicity to anakinra based on these data. The simultaneous application of corticosteroids and anakinra make it difficult to separate the response between anakinra and corticosteroids. Nevertheless, it is clear that anakinra did not have an immediate effect in terms of improving neurological toxicities; it is possible that it had minimal effects or that it modestly shortened the timecourse of neurological toxicities. Due to the fact that anakinra has become routine clinical management in patients experiencing severe CRS and/or ICANS, comparative data of patients who did not receive anakinra are not available. A weakness of this study is the absence of such a comparator group. Future studies to evaluate the impact of anakinra in the management of CAR T-cell toxicity will be necessary, especially regarding neurological toxicities. Although retrospective, the fact that these improvements in clinical and correlative indices were temporally associated with anakinra administration suggests a potential role for IL-1R antagonism in the management of CAR T-mediated toxicities. Here, the decision to use three daily subcutaneous doses was made in the absence of clear data to guide dosage, route of administration and duration of treatment, but generally following the (hope-filled) concept that the effect of anakinra would be rapid and immediate, as was observed when tocilizumab was first used. In our prospective phase II, single-center study using anakinra prophylactically, we chose the same dose and route of administration, given the availability of prefilled syringes, but for a longer duration.

Although limited in number, retrospective and without a comparator group, it is encouraging that we observed an ORR of 55% with a 6- and 12 month OS of 36% and 29% respectively. These data are in line with previous reports suggesting that patients who require aggressive and prolonged steroid use may have inferior outcomes.^6 25^ It is unclear whether the need for such steroid use is a surrogate for high risk factors such as high tumor burden, inflammatory microenvironments and T-cell fitness/expansion, or causative, when examining these inferior outcomes. A weakness of this study in this context includes a missing parameter for tumor burden such as the largest lesion diameter of each patient. This could not be collected due to the retrospective nature of the study. Prospective studies using alternative steroid sparing approaches are needed to better understand the impact of toxicity interventions on clinical outcomes with respect to the underlying disease.

In summary, we have observed that anakinra is associated with improved clinical and laboratory indices of inflammation and modulated cytokine levels in patients who developed corticosteroid-refractory ICANS with or without CRS after treatment with CD19 CAR T-cell therapy, but the effects on correlates of CRS were easier to determine than the effects on neurological toxicity. Our data are limited by their retrospective and correlative nature, but support ongoing studies of anakinra for treatment and prevention of CRS and ICANS. To further evaluate the role of anakinra in CAR T toxicity management, we have opened a phase II single-center study to investigate the effect of anakinra prophylaxis for the prevention of CRS and ICANS in relapsed or refractory non-Hodgkin's lymphoma patients treated with axicabtagene ciloleucel.

## Data Availability

Data are available upon reasonable request.
